# Risk factors associated with post-weaning diarrhoea in Austrian piglet-producing farms

**DOI:** 10.1186/s40813-023-00315-z

**Published:** 2023-05-11

**Authors:** Renzhammer René, Vetter Sebastian, Dolezal Marlies, Schwarz Lukas, Käsbohrer Annemarie, Ladinig Andrea

**Affiliations:** 1 Department for Farm Animals and Veterinary Public Health, University Clinic for Swine, University of Veterinary Medicine, Veterinärplatz 1, Vienna, 1210 Austria; 2 Unit of Veterinary Public Health and Epidemiology, Institute of Food Safety, Food Technology and Veterinary Public Health, University of Veterinary Medicine, Veterinärplatz 1, Vienna, 1210 Austria; 3 Platform for Bioinformatics and Biostatistics, Department of Biomedical Sciences, University of Veterinary Medicine, Veterinärplatz 1, Vienna, 1210 Austria

**Keywords:** Post-weaning diarrhoea, PWD, Colistin, Zinc, Horseradish, Probiotics, Management, Housing, Hygiene

## Abstract

Post-weaning diarrhoea (PWD) is a frequent, multifactorial disease of piglets leading to increased mortality rates and high economic losses. Due to the emergence of multi-resistant *Escherichia coli* isolates and the ban of zinc oxide (ZnO) in the EU since June 2022, alternative measures to prevent PWD are urgently needed. While an abundance of feed supplements is described to prevent PWD, there are hardly any studies reflecting the current situation of PWD in the field. Thus, we aimed to identify differences in management practices, housing and feeding strategies between farms with PWD and farms without PWD. Data were personally collected using a semi-structured questionnaire in 257 Austrian piglet-producing farms. Farms with PWD in more than 10% of all weaned groups within twelve months prior to data collection were defined as case farms (n = 101), while the remaining 136 farms were defined as control farms. Data from 237 farms and 69 explanatory variables were analysed via penalized binary logistic regression using elastic-net in 100 different splits into randomly selected training and test datasets (80:20). Treatment with ZnO and/or colistin (136 farms) was negatively associated with PWD in all splits and had the biggest estimated absolute log odds ratio out of all tested variables. Implementation of an all-in/all-out system in the nursery units and administration of probiotics or horseradish also had preventive effects in most splits (≥ 97%). A higher number of feeding phases for piglets within the first seven weeks of life and housing on fully slatted floors was associated negatively with the occurrence of PWD as well in > 95% of all splits. PWD was more likely to occur on farms having problems with neonatal diarrhoea or postpartum dysgalactia syndrome. While our data demonstrate that treatment with ZnO or colistin had the biggest statistical effect on PWD, we were able to identify other preventive measures like supplementation with probiotics or horseradish. Since implementation of all-in/all-out measures and fully slatted floors were also negatively associated with the occurrence of PWD on visited farms, we assume that reduction of bacterial load by the implementation of simple hygiene measures are still crucial to prevent PWD.

**Supplementary Information:**

The online version contains supplementary material available at 10.1186/s40813-023-00315-z.

## Background

Post-weaning diarrhoea (PWD) occurs within the first two weeks after weaning of piglets and can lead to high economic losses due to increased mortality rates, reduced growth rates and increased costs for treatment [1]. Moreover, PWD is also considered as one of the most frequent diseases in pig production worldwide. Although PWD is a multifactorial disease, ubiquitous enterotoxigenic *Escherichia* (*E.*) *coli* (ETEC) play a pivotal role in the pathogenesis of PWD [1, 2]. To a lesser extent other pathogens like enteropathogenic *E. coli* (EPEC) and *Salmonella* spp. are also potential infectious causatives of PWD [1, 3, 4]. Furthermore, the impact of rotavirus A and C on PWD is currently discussed controversially [5]. Nevertheless, feeding, management and housing conditions play a crucial role in the pathogenesis of PWD. In particular, the abrupt transition from highly digestible milk to less-digestible solid feed consisting of mainly plant based proteins, complex carbohydrates and several anti-nutritional factors overburdens the intestinal tract of piglets, which are usually weaned between their 21st to 28th day of life [2, 6]. Most notably, weaning is associated with anorexia of piglets within the first two days after weaning leading to villous atrophy and crypt hyperplasia in the small intestine [7–9]. Besides the reduction of nutrient absorption due to the villous atrophy, there is evidence of a decline in brush-border enzymes leading to inferior protein digestibility and malnutrition [9]. While low gastric pH is a crucial defence mechanism against ETEC, pH values in the stomach and small intestine increase significantly after weaning and consequently facilitate intestinal colonization with ETEC [10]. Higher gastric pH values after weaning are the result of a reduced HCl production due to anorexia, higher amounts of less-digestible proteins in the feed and decreased production of lactic acid produced from lactose [6, 11, 12]. Furthermore, deprivation of milk and increased pH values cause significant shifts in the intestinal microbial composition characterized by a decrease of gram-positive bacteria like *Lactobacillus* spp. and *Bifidobacterium* spp. and an increase of *Enterobacteriaceae* [13]. Besides multiple nutritional causes of PWD, the process of weaning significantly increases the level of immunosuppressive cortisol due to several stressors like separation from the sow, transfer to new pens and mixing of different litters [14].

While antimicrobials are widely applied to treat piglets with PWD, numerous reports on high antimicrobial resistance rates of porcine *E. coli* within the last few years emphasize the difficulties to choose adequate treatment of diseased piglets [15–17]. Thus, veterinarians frequently apply colistin (polymyxin E) for oral treatment of piglets with PWD, since resistance rates of porcine *E. coli* against colistin are considered to be low [18, 19]. However, since colistin is crucial to treat humans affected by multi-resistant gram-negative bacteria, its use to treat animals is under debate [18].

As an alternative to antimicrobials, zinc oxide (ZnO) has been frequently applied in swine farms worldwide to treat piglets with PWD [20]. In general, zinc is a pivotal trace element for pigs, as it is essential for multiple biochemical processes [21]. Therefore, commercially available feed contains zinc at a concentration of 150 ppm, which is defined as the maximum nutritional concentration. First beneficial effects including a decreasing incidence of PWD can be observed, if piglets are treated with a concentration of 1,000 ppm ZnO [22]. In contrast to antimicrobial substances like colistin, there is evidence that ZnO does not act with a specific mechanism against *E. coli*, but rather improves gut health, digestion and the immune system of treated piglets in various ways. Described beneficial mechanisms include an increased expression of occludin, increased levels of superoxide dismutase and a reduction of pro-inflammatory cytokines [20, 23–25]. The combination of anti-oxidative, anti-inflammatory and immune stimulating mechanisms of ZnO might lead to lower incidences of intestinal inflammation and diarrhoea. Furthermore, treatment with ZnO (2,500 ppm) can also improve digestibility of nutrients due to increased production of amylase, carbopeptidase A, chymotrypsin, trypsin and lipase [26].

On the other hand, there is evidence that treatment of piglets with ZnO (2,500–3,000 ppm) for 21 days increases the prevalence and persistence of methicillin-resistant *Staphylococcus aureus* [27, 28] and antimicrobial resistance rates of porcine *E. coli* towards ampicillin, tetracycline and trimethoprim-sulfonamide [29–31]. In particular, environmental concerns led to the ban of ZnO in the European Union [32]. A prospective hazard analysis emphasizes the potential danger of ZnO applied in livestock for soil and ground water in the Netherlands, Flanders, north-western Germany and Denmark [33]. After the conduction of the latter study, the European Medicines Agency (EMA) concluded that disadvantages of ZnO usage outweigh its benefits leading to the EU-wide ban of all licensed medicinal products containing ZnO with a concentration beyond 150 ppm [32]. However, applicable alternatives for ZnO are yet to be identified. High antimicrobial resistance rates of porcine *E. coli* isolates, the goal of the European Commission to reduce usage of antimicrobial substances in livestock and the ban of ZnO since 26th of June 2022 emphasize the urgent need for applicable strategies to prevent PWD reliably. Although a plethora of potential alternatives to ZnO is described, ZnO has still been applied in most swine farms in Austria and other EU countries until 26th of June 2022, emphasizing the lack of a strategy to successfully prevent the occurrence of PWD.

Late weaning, reduction of crude protein and increase of crude fibre in the feed are well described methods to reduce the incidence of PWD [34–38]. Numerous feed additives have already been proposed as potential alternatives to ZnO [6, 20]. Feed additives, which potentially reduce the incidence of PWD include for example organic acids [39–41], probiotics [42–45], prebiotics [46, 47], essential oils and other plant based substances [48, 49], exogenous enzymes [50, 51], dehydrated porcine plasma [52], yeast [53] and antimicrobial peptides [54, 55]. However, while most studies on alternative strategies to reduce the usage of antimicrobial substances focus on feed additives, only a few studies report on the impact of management, housing conditions and biosecurity [56]. Due to the fact, that PWD is a multifactorial disease, it is crucial to evaluate all parameters, which potentially prevent or provoke the onset of diarrhoea. Furthermore, since most studies on prevention of PWD were conducted under experimental settings [56], they cannot reflect the situation in the field appropriately.

Thus, we aimed to reflect the frequency of PWD and the use of ZnO and/or colistin treatment in Austrian piglet-producing farms. Furthermore, we wanted to identify differences of management practices, housing conditions, feeding strategies and biosecurity measures between farms with PWD and farms without PWD.

## Results

### Descriptive analysis

In all 101 case farms (42.6%) first symptoms of PWD appeared between the first and 8th day post weaning (mean = 3.8 days post weaning). Out of all 142 farms with regular treatment against PWD, 132 farms applied products containing ZnO, 56 of them in combination with colistin. While products containing only colistin were applied in four farms, marbofloxacin and gentamicin were used in two and four farms respectively. Means of most numerically coded variables like age at weaning, crude protein, crude fibre, feeding space per piglet, number of piglets per drinking spot, square meters per piglet and piglets per pen did not vary among case and control farms (Table 1).

**Table 1:** Exploratory arithmetic means for numerically coded explanatory variables in the elastic-net model from 237 farms

			Age at weaning (days)	Number of feeding phases	Crude Protein g/kg DM	Crude Fiber g/kg DM	Feeding space per piglet (cm)	Number of piglets per drinking spot	Square meters per piglet	Piglets per batch
PWD	No	Mean	26.7	3.2	165.7	43.6	8.4	13.5	0.3	43.7
	Yes	Mean	26.9	2.9	164.9	45.2	8.1	15.4	0.3	44.7

PWD: post-weaning diarrhoea; DM: dry matter

Since PWD was perceived as a general herd problem, 97 farmers (40.9%) stated to have supplemented ZnO and/or colistin to the feed of at least 50% of all batches of weaned piglets and did not observe PWD within twelve months prior to data collection (Table 2). Further 45 farmers (19.0%) claimed to observe PWD regularly despite treatment with ZnO or colistin. PWD occurred on 56 other farms (23.6%), neither administering ZnO nor colistin at weaning. In 39 farms (16.5%) PWD was no problem and therefore administration of ZnO or colistin was not necessary at all. The occurrence of PWD was lower in farms supplementing probiotics (27%) compared to those without supplementation of probiotics (47%). Administered probiotics belonged to the genera *Pediococcus* (n = 19), *Lactobacillus* (n = 17), *Enterococcus* (n = 9) and *Streptococcus* (n = 3). Out of 32 farms adding horseradish to the feed after weaning, three quarters did not have problems with PWD (Table 2). The effect of other supplementary substances based on plants could not be evaluated individually, as declaration of content was rarely provided. Thus, other feed additives based on plants were grouped together to the variable “herbs”.

**Table 2:** Occurrence of PWD and number of observations for categorical variables from 237 farms

		PWD				PWD	
		No	Yes			No	Yes
**Treatment**	No	39	56	**Lactic acid**	No	83	64
	Yes	97	45		Yes	53	37
**Neonatal diarrhoea**	No	56	24	**Benzoic acid**	No	85	56
	Yes	80	77		Yes	51	45
**Postpartum dysglactia syndrome**	No	76	41	**Sorbic acid**	No	98	79
	Yes	60	60		Yes	38	22
**Rearing of own gilts**	No	78	55	**Coke, phosphoric acid**	No	110	75
	Yes	58	46		Yes	26	26
**Nurse sows**	No	95	62	**Electrolytes, glucose**	No	121	87
	Yes	41	39		Yes	15	14
**Cross fostering**	No	22	10	**Apple pomace**	No	123	92
	Yes	114	91		Yes	13	9
**Weaning in farrowing units**	No	98	74	**Dairy products**	No	122	96
	Yes	38	27		Yes	14	5
**Grouping of weaned piglets**	Sorted by size	63	40	**Rock salt**	No	122	81
	Litter wise	73	61		Yes	14	20
**Automatic feeding system**	No	96	80	**Yeast**	No	93	81
	Yes	40	21		Yes	43	20
**Stimulation of feed intake**	No	98	71	**Tannins**	No	119	92
	Yes	38	30		Yes	17	9
**Weaner diet before weaning**	No	33	23	**Anticoccidials**	No	93	66
	Yes	103	78		Yes	43	35
**Main starch source**	Corn	44	34	**Water source**	Mains water	34	26
	Barely	92	67		Well water	102	75
**Wheat, rye, triticale**	No	75	67	**Calcareous water**	No	54	46
	Yes	61	34		Yes	82	55
**Main protein source**	Plant	122	93	**Disinfection of water pipes**	No	110	85
	Animal	14	8		Yes	26	16
**Main soy processing method**	Heated	74	52	**Looped water distribution system**	No	119	95
	Extracted	62	49		Yes	17	6
**Potato protein**	No	43	31	**Rinsing of water pipes**	No	65	46
	Yes	93	70		Yes	71	55
**Linseed, chicory**	No	80	60	**Diameter of water pipes in nursery**	1/2 inch	116	79
	Yes	56	41		> 1/2 inch	20	22
**Oats**	No	53	47	**Drinkers**	Only nipples	69	49
	Yes	83	54		Also bowls	67	52
**Wheat bran**	No	70	47	**Rodent burden**	Low	77	44
	Yes	66	54		High	59	57
**Cellulose**	No	77	59	**Enrichment material**	Only wood/chains	73	54
	Yes	59	42		Also straw/ropes	63	47
**Fibre in creep feed**	No	73	67	**Microclimate zones**	No	96	75
	Yes	63	34		Yes	40	26
**Milk substitution**	No	76	57	**Heating of nursery before weaning**	No	25	28
	Yes	60	44		Yes	111	73
**Muesli**	No	100	80	**All-in/all-out in nursery units**	No	73	72
	Yes	36	21		Yes	63	29
**Commercial peat**	No	111	92	**Hospital pens**	No	53	46
	Yes	25	9		Yes	83	55
**Probiotics**	No	101	88	**Fully slatted floors**	No	70	36
	Yes	35	13		Yes	66	65
**Horseradish**	No	112	93	**Plastic or metal floors**	No	92	53
	Yes	24	8		Yes	44	48
**Herbs**	No	119	89	**Regular cleaning and disinfection**	No	60	36
	Yes	17	12		Yes	76	65
**Formiate, acetate, or propionate**	No	41	35	**Usage of detergents**	No	66	48
	Yes	95	66		Yes	70	53
**Citric acid**	No	78	56	**Equipment only for nursery units**	No	81	61
	Yes	58	45		Yes	55	40
**Lauric acid**	No	120	90	**Hygiene lock**	No	105	85
	Yes	16	11		Yes	31	16

PWD: post-weaning diarrhoea

### Elastic-net results

Variables associated with PWD in at least 60% of elastic-net splits (equivalent to a minimum of 60% of non-zero penalized logit estimates for the variable across 100 different splits into training and test data), were considered to have at least some impact on the occurrence of PWD. A potential influence on the output of PWD could be determined for 22 out of 69 analysed explanatory variables using elastic-net (Table 3). Eight variables (treatment, neonatal diarrhoea, all-in/all-out system, probiotics, postpartum dysgalactiae syndrome (PDS), horseradish, number of feeding phases and solid floors) were associated with the occurrence of PWD in ≥ 95% of all splits indicating a high impact on the incidence of diarrhoea (Table 3). Regular administration of ZnO and/or colistin, implementation of an all-in/all-out system in the nursery units, usage of probiotics and administration of horseradish had negative estimated log odds ratios in ≥ 97% of all splits demonstrating a potential preventive effect on the occurrence of PWD. The number of feeding phases for piglets from farrowing until the 21st day after weaning and housing on fully slatted floors were also negatively associated with the occurrence of PWD in at least 95% of all splits. Log odds ratios were positive for neonatal diarrhoea and PDS in ≥ 98% of all splits demonstrating that farms having problems with neonatal diarrhoea or PDS were also more likely to have problems with PWD.

**Table 3:** Variables associated with PWD in over 60% of all training-test data splits using elastic-net (n = 22/69)

Variable	5% Percentile	Median		95% Percentile	% Non zero	Reduced chance of PWD	
**Treatment**	–**1.40**	–**1.07**	–**0.71**		**100**		**Yes**
**Neonatal diarrhoea**	**0.15**	**0.43**	**0.73**		**100**		**No**
**All in-all out in nursery units**	–**0.82**	–**0.51**	–**0.19**		**99**		**Yes**
**Probiotics**	–**0.99**	–**0.62**	–**0.18**		**99**		**Yes**
**PDS**	**0.05**	**0.36**	**0.68**		**98**		**No**
**Horseradish**	–**1.03**	–**0.66**	–**0.24**		**97**		**Yes**
**Number of feeding phases**	–**0.38**	–**0.20**	–**0.03**		**96**		**More phases**
**Solid floors**	**0.02**	**0.35**	**0.65**		**95**		**No**
Commercial peat	–0.94	–0.54	0.00		94		Yes
Rodent burden	0.00	0.22	0.51		90		Low
Nurse sows	0.00	0.28	0.53		90		No
Plastic or metal floors	–0.60	–0.30	0.00		89		Yes
Dairy products	–0.74	–0.30	0.00		86		Yes
Looped water distribution system	–0.64	–0.23	0.00		82		Yes
Rock salt	–0.67	–0.21	0.00		82		Yes
Coke / phosphoric acid	0.00	0.28	0.61		82		No
Cross fostering	0.00	0.28	0.75		80		No
Regular cleaning and disinfection of nursery units	–0.44	–0.14	0.00		76		Yes
Automatic feeding	–0.55	–0.20	0.00		75		Yes
Yeast	–0.48	–0.16	0.00		73		Yes
Fibre in creep feed	–0.34	–0.07	0.00		69		Yes
Electrolytes, glucose	–0.63	–0.07	0.00		60		Yes

% Non zero: percentage of splits with log odds (ratios) unequal zero; ZnO: zinc oxide; PDS: postpartum dysgalactia syndrome; Variables for which the 5 and 95 percentile of the logit distribution across 100 elastic-net splits did not include zero are marked in bold

**Fig. 1:**
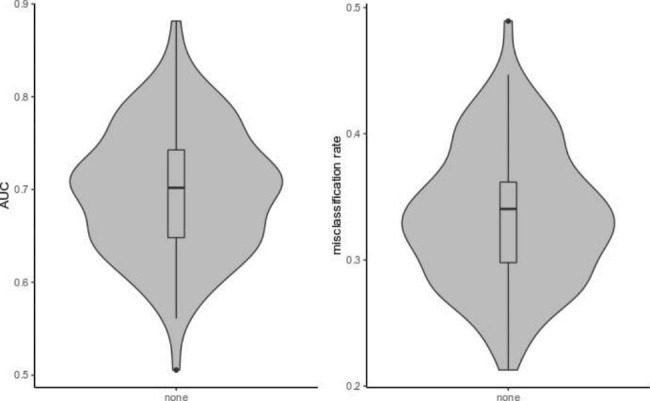
Violin plots for area under the curve (AUC) and misclassification rates across 100 elastic-net models

Out of all 69 variables tested, treatment with ZnO and/or colistin had the biggest absolute median (– 1.07) of the estimated log-odds ratio distribution across 100 elastic-net splits. (Additional file 1). To assess model fit we looked at the area under the curve (AUC) which varied between 0.51 and 0.88. Misclassification rates ranged from 0.21 to 0.49 in the test data across all 100 splits (Fig. 1).

### Variables excluded from statistical analysis

PWD was no problem in eight out of nine farms not offering an additional transition feed between creep feed and weaner diet. Variables on transition feed were excluded from analysis due to collinearity with the number of feeding phases. Neither problems with PWD nor treatment against PWD were reported from any of the six farms supplementing creep feed with seaweed. PWD occurred in one out of six farms using disinfection troughs in front of the nursery units. Out of eight farmers applying a modified live vaccine against ETEC, three claimed that problems with PWD occurred despite vaccination. Supplementation of seaweed, usage of disinfection troughs and vaccination of piglets against ETEC were excluded from analysis because the number of observations for one factor level was < 10 (Additional file 2).

## Discussion

Administration of ZnO or colistin revealed strongest effects on the occurrence of PWD out of all 69 analysed explanatory variables. Similar to the results of an experimental study demonstrating that treatment with ZnO (2,500 ppm) reduced the incidence of diarrhoea by approximately 50% [57], in our study the percentage of farms with PWD not using ZnO was almost twice as high (58.9%) compared to farms with regular administration of ZnO (31.7%). However, 45 farmers reported to observe PWD regularly in nursery pens despite treatment of weaned piglets. Thus, ZnO does not prevent PWD completely, especially if other protective measures might be neglected. On the other hand, our data demonstrate that administration of other feed additives such as probiotics or horseradish can prevent PWD, although effects were less pronounced compared to ZnO. Therefore, we assume that a combination of different management practices along with the administration of other feed additives may compensate at least partially for the administration of ZnO to prevent PWD.

Besides treatment with ZnO and/or colistin, only neonatal diarrhoea was also associated with the occurrence of PWD in all splits, which was not surprising since ETEC expressing F4 fimbriae can cause both, neonatal diarrhoea and PWD [1]. Furthermore, piglets which have already had neonatal diarrhoea might be more susceptible to PWD due to previous damage in the intestines and worse body condition compared to piglets without diarrhoea during the suckling period. Therefore, several approaches to prevent neonatal diarrhoea like sow vaccination against ETEC, *Clostridium perfringens* or rotavirus A, as well as treatment of piglets with anticoccidials might prevent PWD indirectly. However, those variables were excluded from statistical analysis due to collinearity with the occurrence of neonatal diarrhoea. Similar to PWD, PDS is a multifactorial disease with *E. coli* playing a key role in its pathogenesis [58]. Thus, parameters increasing the risk for PDS might also increase the risk of PWD. Moreover, dysgalactia of sows leads to malnutrition and reduced growth rates of suckling piglets resulting in a higher susceptibility to disease.

Despite the fact that good feeding management and choice of beneficial ingredients in the feed are described to be very crucial to prevent PWD [20], the number of feeding phases until 21 days after weaning was the only variable on feeding management which was associated with the occurrence of PWD in at least 95% of all splits. This could be due to the lack of information on quantities of ingredients contained in purchased commercial products. While in most Austrian farms corn, barley and wheat are produced on-site and exact quantities used in feed were available, protein sources were predominantly supplemented with commercial products. Since information on exact quantities of all ingredients contained in each commercial product (n = 96) was not available, precise evaluation of feed ingredients was not feasible. Although we had to exclude most variables on creep feed from statistical analysis due to collinearity, information whether creep feed was offered or not, is still contained in the variable “number of feeding phases”. This could explain why farms with more feeding phases were less likely to have problems with PWD, since it was demonstrated before that administration of creep feed can decrease the risk of PWD [59]. In addition, creep feeding containing fibre had preventive effects in 69% of all splits. While van Hees et al. did not observe significant differences in average daily gain (ADG) between piglets getting low-fibre creep feed and piglets with high-fibre creep feed after challenge with ETEC [60], Choudhury et al. demonstrated a reduced incidence and duration of PWD in piglets fed fibrous pre-weaning diet compared to the control group receiving only milk [61].

Despite the fact that reduction of crude protein and increase of crude fibre are widely known strategies to prevent PWD [36], we did not observe any differences of both variables among case and control farms. Our results also go in line with observations of another investigation [62]. In most studies evaluating the effect of dietary protein level, incidence of diarrhoea was higher in piglets receiving feed with protein levels ranging from 20 to 26% compared to groups receiving 16–19% crude protein in feed [34–38, 63, 64]. Therefore, we assume that the level of protein did not have an influence on the occurrence of PWD in our data, since protein levels of most analysed farms were already comparably low (16–17%). Hence, reduction of crude protein and increase of crude fibre in the diet of weaned piglets alone might not be sufficient to prevent PWD under those circumstances. Albeit in our study farms with automatic feeding in the nursery units had less problems with PWD compared to farms feeding piglets manually, no differences between automatic and manual feeding were observed in a similar study conducted in Finland [65].

In contrast to feeding management and feed composition, several variables on feed additives (8/18) were associated with PWD in at least 60% of all splits. Out of all analysed feed additives, supplementation of probiotics or horseradish had the strongest effects on the occurrence of PWD (Table 3). While strains of *Lactobacillus* spp., *Bifidobacterium* spp. and *Streptococcus* spp. are administered most frequently in pig production worldwide [66], *Bifidobacterium* spp. was not administered in a single analysed farm. Administration of probiotics might have multiple effects on the intestines, similar to treatment with ZnO, including an increase of villus heights in the jejunum [67] and decreased levels of tumour necrosis factor alpha [67, 68] and interleukin-1 beta [68]. Horseradish (*Armoracia rusticana*) is a plant commonly grown in Styria and has not been described to have preventive properties against PWD so far. Potential explanations why farms administering horseradish were less likely to have problems with PWD could be provided by confirmed antimicrobial properties of mustard essential oils, which are contained in horseradish [69, 70]. Unique effects of the administration of horseradish on the prevalence of PWD are yet to be evaluated in experimental trials or additional field studies.

Humic acids contained in peat [71] could explain why the majority of farms administering peat reported to have no problems with PWD (25/34). Administration of 2,000 ppm sodium humate to 24 piglets resulted in significantly higher ADG and decreased levels of pro-inflammatory cytokines compared to the untreated control group [72]. Since offering pig peat permits expression of natural behaviour and reduces fighting at weaning, it might also decrease stress levels of piglets and further decrease the probability of PWD [73]. Nevertheless, peat is a frequently described hazard for the introduction of pathogens [74, 75]. Therefore, offering peat to prevent PWD should be reconsidered based on biosecurity protocols. Since whey protein and casein are well digestible for three to four week old piglets, supplementation of various dairy products, which was also negatively associated with the occurrence of PWD in 86% of all splits, could be considered as a preventive measure against PWD as well [76].

There is currently no evidence that coke could also prevent PWD. In contrast, our data demonstrate that farms administering coke to piglets were more likely to have problems with PWD. Phosphoric acid, an ingredient of coke is an inorganic acid and has never been described to prevent PWD [77]. We assume that administration of coke to piglets might be linked to the fact that coke is still used as a home remedy against diarrhoea. This old wives’ tale could be related to the fact that citric acid was used instead of phosphoric acids in previous formulations of coke. In comparison to phosphoric acid, it has already been demonstrated that supplementation of citric acid can prevent diarrhoea evidently [41, 77, 78].

PWD was also less likely to occur in farms administering live yeast and electrolytes, but effects were weaker compared to administration of probiotics or horseradish. Capability of yeast (*Saccharomyces cerevisiae*) to reduce incidence of PWD and shedding of *E. coli* has already been demonstrated in experimental studies [53, 79]. Additionally, similar to ZnO it was demonstrated that administration of yeast decreased serum concentrations of pro-inflammatory cytokines [80]. Application of glucose or electrolytes could generally temper symptoms of PWD [81].

Pronounced effects of variables like all-in/all-out system in the nursery or the type of floor in most splits highlight the importance of general management measures in order to prevent PWD (Table 3). Consequent compliance of an all-in/all-out system in the nursery units can reduce the frequency of PWD, probably due to a reduction of pathogen transmission between piglets of different age groups [82]. While implementation of an all-in/all-out system proved to reduce the prevalence of various enteropathogens [83–85], it had no significant effect on the prevalence of ETEC in a Canadian study [62]. In addition to an all-in/all-out system, other measures reducing pathogen transmission between individuals might also be beneficial to prevent PWD. One example could be to avoid or reduce cross fostering, as implementation of this management practice was associated positively with the occurrence of PWD in 80% of all splits.

Despite discussions on fully slatted floors in pig production in the context of animal welfare, our data emphasize the advantages of fully slatted floors in the context of hygiene measures, since PWD was less likely to occur in farms keeping piglets on fully slatted floors. Due to easier cleaning and disinfection and the quick removal of faeces through the slats, bacterial load is considered to be lower in pens with fully slatted floors compared to solid floor pens [86, 87]. On the other hand, diarrhoea can be observed more easily in pens with solid floor. Similar to our results, Lozano et al. demonstrated that piglets housed on fully slatted plastic floors were less likely to develop PWD than piglets housed on concrete floors [88]. Berrocoso et al. demonstrated that husbandry of weaned piglets under optimal hygienic conditions including regular cleaning with detergents and disinfection resulted in significantly lower incidence of PWD and better ADG of weaned piglets [89]. Since investigated farms which regularly cleaned and disinfected nursery units were less likely to have problems with PWD, our results emphasize that hygienic measures are crucial to decrease ETEC burden and shall not be neglected in order to prevent PWD.

Besides environment, drinking water and water pipes could be another reservoir of ETEC [90]. Due to the fact that looped water distribution systems have less dead endings compared to branched water distribution systems, accumulation of sediments and biofilm formation is less likely [91]. Thus, infections with ETEC via contaminated water coming from water pipelines with branched endings could be more likely, since ETEC are frequently recovered from biofilms in water pipes [90, 92].

In addition, rats and mice can also act as a vehicle for introduction of various *Enterobacteriaceae* like *Salmonella* spp. [93, 94]. Even though there are currently no data on the relation between rodent burden and incidence of PWD, our results emphasize that high rodent burden could presumably lead to increased transmission rates of ETEC and higher bacterial loads. Thus, strict measures against rodents might potentially decrease the probability of occurrence of PWD in swine farms.

Data collection through questionnaires heavily depends on perspectives and honesty of participants. Thus, bias due to dishonesty, subjective assessments of farmers and recall bias cannot be excluded. However, in order to reduce further bias related to subjectivity, data were always collected by the same person, who visited each farm to ensure consistent quality of collected data. Especially, recall bias for classification into case and control farms might have been high due to difficulties to estimate the number of weaned batches having problems with PWD. Additionally, farmers spending more time in the stables might be more likely to observe PWD. Furthermore, separate analysis for ZnO and colistin was not feasible, since products containing both substances were administered in numerous farms. Categorization of the variable treatment into “yes” or “no” for farms treating at least 50% of all batches of weaned piglets with antibiotics other than colistin, was done based on the results of a previous study with *E. coli* isolates recovered from Austrian swine farms [16]. However, antimicrobial resistance testing was not performed for *E. coli* from the visited farms in the current study.

Since an abundance of factors contributes to the pathogenesis of PWD, the number of variables could not be further limited without deleting pivotal parameters. Thus, elastic-net model was chosen to assess the influence of each variable for the output of PWD. While elastic-net is able to handle datasets with numerous predictors, it cannot deliver p-values. Therefore, we decided to renounce p-values. On the other hand, by using this method we were able to identify more variables with an influence on the output of PWD compared to previous similar analyses on PWD [62, 65]. McFadden pseudo R² accounting for 0.47 implicates that our dataset was able to partially explain the observed variance. However, since PWD is a complex, multifactorial disease the unexplained variance was no surprise. Further, certain factors influencing the output of PWD might have not been included in the questionnaire or during data processing.

## Conclusions

Out of all analysed variables, treatment with ZnO and/or colistin was most preventive against PWD. Nevertheless, we were able to identify alternative preventive measures like supplementation of probiotics or horseradish, which might contribute to a lower incidence of PWD. However, their effects need to be evaluated further. We could also demonstrate that the occurrence of PDS and neonatal diarrhoea was associated positively with the occurrence of PWD on visited farms. Since implementation of all-in/all-out measures and fully slatted floors were clearly negatively associated with the probability of PWD, reduction of bacterial load by the implementation of simple hygiene measures might still be pivotal to prevent PWD.

## Methods

### Data collection

A semi-structured questionnaire was used in order to receive sufficient information from Austrian piglet producing farms (Additional file 3). In total, the questionnaire consisted of 156 questions concerning (a) general farm data, (b) suckling piglet period, (c) weaning management, (d) housing of piglets, (e) feed & feeding management, (f) feed additives & other preventive methods, (g) water supply, (h) hygiene and biosecurity, and (i) occurrence of PWD, including treatments with antimicrobials or ZnO (Table 4).

**Table 4:** Number of questions in each category included in the questionnaire

Category	Number of questions
General farm data	14
Suckling piglet period	19
Weaning management	12
Housing of piglets	13
Feed & feeding management	18
Feed additives & other preventive methods	9
Post-weaning diarrhoea & treatments	28
Water supply	15
Hygiene and biosecurity	28

### Study design & setting of the study

Pig farmers were first contacted with the help of their respective herd veterinarian (n = 40) and asked to participate in the study. Farmers agreeing to participate signed a consent form and were always interviewed on the farm by the same person in a standardised way. Data were collected from 257 piglet-producing farms throughout Austria from May 2020 to March 2021 (Tables 5 and 6). The estimated time for the survey including measuring the size of two pens in the nursery units accounted for approximately 75 min.

**Table 5:** Regional distribution and farming system of the 257 visited farms

	Number of visited farms	
**Federal state**	Upper Austria	85
	Lower Austria	81
	Styria	70
	Carinthia	16
	Burgenland	5
**Farming system**	Conventional farming	242
	Organic farming	15

**Table 6:** Farm characteristics of all 257 visited farms

	Median	Minimum	Maximum
**Number of sows**	80	12	2300
**Weaned piglets/sow/year**	25.2	14.8	39.5

### Data processing

All data (257 farms with 225 variables) were transferred to Microsoft® Excel and analysed anonymously. In order to represent the majority of piglet-producing farms in Austria, organic farms (n = 15), farms with less than 14 sows (n = 3) and farms with over 500 sows (n = 2) were excluded from further analysis. All variables with missing data were excluded from statistical analysis (n = 39). Those variables predominantly derived from answers of sub-questions, which were NA by default depending on the answer to the previous question (30/39). Several variables, like those on general farm data and vaccination protocols against pathogens associated with respiratory disease were excluded as well (n = 46), since they were considered as irrelevant for PWD. Categorical variables were also excluded if the number of observations for one factor level was below ten (n = 21). To avoid exclusion of too many variables, certain values (i.e.: metal floors & plastic floors) or variables (i.e.: coke & phosphoric acid) were combined, if the combination was biologically reasonable. The variable treatment was defined as yes, if either ZnO with a concentration beyond 1,000 ppm or colistin were supplemented at weaning for at least 50% of all batches of a farm within the year prior to data collection (n = 136). Six additional farms applying marbofloxacin (n = 2) or gentamicin (n = 4) instead of ZnO or colistin were also included in the treatment group. For the sake of simplicity, treatment with marbofloxacin and gentamicin are not explicitly mentioned further. The variable treatment was defined as “no” for the remaining farms including farms regularly applying aminopenicillins (n = 10), tetracyclines (n = 3) or macrolides (n = 3) after weaning, due to high resistance rates of ETEC towards those substances [16]. Nevertheless, it is likely that those antibiotics were predominantly applied to treat piglets against other diseases. Questions on PWD and treatment aimed to reflect the frequency of PWD and ZnO and colistin usage in Austrian swine farms. Thus, besides the variable treatment, variables from that category were also excluded from further analysis (n = 27). Details on complete data processing are provided in Supplementary File 2.

### Definition of case and control farms

If the farmer stated to have observed at least one piglet with diarrhoea in at least 10% of all weaning groups in the first two weeks after weaning within twelve months prior to data collection, the farm was categorized as case farm (101/237).

### Statistical analysis

All statistical analyses were performed in R version 4.0.2 [95]. The remaining data (237 farms and 92 explanatory variables) were imported into the statistics software R and tested for collinearity. In case of collinearity (variance inflation factor > 3) calculated in package *car v3.0.12* [96], applying function *vif* on a linear model containing all explanatory variables, the variable with a lower expected biological relevance was excluded (n = 23) [58]. In the end, 69 variables remained in the final data set for statistical analysis (Tables 1 and 2). Since application of a standard (generalized) linear model with 237 farms and 69 explanatory variables would have led to a severe overfitting problem, data were analysed via penalized binary logistic regression using elastic-net implemented in package *glmnet v4.1.4* [97]. Elastic-net can be thought of as a combination of shrinkage (Ridge Regression) and variable selection (LASSO) methodology. The binary response was coded “0” for control farms and “1” for case farms. The function *createDataPartition* from package *caret v6.0-92* [98] was applied to split questionnaires from 237 farms into a randomly selected independent training- and test dataset comprising of 80% and 20% of farms, respectively.

Cross validation was used to fine-tune the elastic-net parameters in the training data by applying function *cv.glmnet* with option family=”binomial”, standardize = T and maxit = 500,000. Model performance was assessed in the independent test data applying function *assess.glmnet* using option family="binomial” and s="lambda.min”. “lambda.min” contains stored regularization parameter giving the minimum mean cross-validated error in the training data. Due to sample size limitations, we noticed that different seeds used for splitting the data into training and test data, resulted in somewhat different variable selections and size of estimated logits. Thus, we decided to create 100 different random splits (training data:test data = 80:20) using function *set.seed(i)* in a loop over our elastic-net analysis, conducted as described above. Afterwards, model performance was assessed via the AUC metric and misclassification rates summarized and visualized as violin-plots (Fig. 1). Furthermore, we report size of penalized estimated coefficients on a logit level and the number of times out of 100 (i.e.%) an explanatory variable was selected for the model (Supplementary File 1). Negative estimated coefficients on logit level, imply an odds ratio < 1, and the variable is negatively associated with the occurrence of PWD or has a preventive effect towards PWD. Variables with positive coefficients imply an odds ratio > 1 and are positively associated with the occurrence of PWD increasing the chance of PWD on a farm.

## Electronic supplementary material

Below is the link to the electronic supplementary material.

Additional file 1: Title: Logit distribution of all 69 tested explanatory variables across 100 elastic-net splits. Description: Results from elastic net analysis including percentiles (p5, p10, p25, p50, p75, p90, p95) and % non zero (percentage of splits with log odds (ratios) unequal zero) of estimated logits from all 69 explanatory variables across 100 elastic-net splits.

Additional file 2: Title: Data processing. Description: Complete data processing including the pathway from the original question provided in Additional file 3 to the final variables included in the statistical analysis. Additional file 2 includes English translations of all 156 original German questions. Information on variable type and reason for exclusion of the respective variable are also provided in Additional file 2.

Additional file 3: Title: Questionnaire. Description: Original questionnaire including all 156 questions in German language.

## Data Availability

All collected data are available as supplementary material.

## Abbreviations

PWD: Post-weaning diarrhoea
*E. coli*: *Escherichia coli*
ETEC: Enterotoxigenic *E. coli*
EPEC: Enteropathogenic *E. coli*
ZnO: Zinc oxide
PDS: Postpartum dysgalactia syndrome
ADG: Average daily gain
