# Correction: Risk factors associated with post-weaning diarrhoea in Austrian piglet-producing farms

**DOI:** 10.1186/s40813-023-00322-0

**Published:** 2023-06-08

**Authors:** René Renzhammer, Sebastian Vetter, Marlies Dolezal, Lukas Schwarz, Annemarie Käsbohrer, Andrea Ladinig

**Affiliations:** 1 Department for Farm Animals and Veterinary Public Health, University Clinic for Swine, University of Veterinary Medicine, Veterinärplatz 1, 1210 Vienna, Austria; 2 Unit of Veterinary Public Health and Epidemiology, Institute of Food Safety, Food Technology and Veterinary Public Health, University of Veterinary Medicine, Veterinärplatz 1, 1210 Vienna, Austria; 3 Platform for Bioinformatics and Biostatistics, Department of Biomedical Sciences, University of Veterinary Medicine, Veterinärplatz 1, 1210 Vienna, Austria

**Correction : Porcine Health Management (2023) 9:20**

10.1186/s40813-023-00315-z

Following the publication of the original article [1], the authors identified an error in the author group. The given names and family names were erroneously transposed.

The incorrect authors’ names are: Renzhammer René, Vetter Sebastian, Dolezal Marlies, Schwarz Lukas, Käsbohrer Annemarie and Ladinig Andrea.

The correct authors’ names are: René Renzhammer, Sebastian Vetter, Marlies Dolezal, Lukas Schwarz, Annemarie Käsbohrer and Andrea Ladinig.

The author group has been updated above and the original article [1] has been corrected.
