# Supplementary material for: Risk factors associated with post-weaning diarrhoea in Austrian piglet-producing farms
Source: Porcine Health Manag. 2023 May 11;9:20. doi: 10.1186/s40813-023-00315-z (PMC10176918; doi:10.1186/s40813-023-00315-z)
Supplement: Supplementary file 3 — Additional file 3: Title: Questionnaire. Description: Original questionnaire including all 156 questions in German language. [file 40813_2023_315_MOESM3_ESM.pdf]

1. Allgemeine Betriebsdaten

1. Höchste Ausbildung des Betriebsleiters (inkl. Zweig): _______________(____________)

2. Jahre der Berufserfahrung:_______

3. Betreuungstierarzt:_____________________

4. **Betriebsart** ☐Konventionell ☐Biologisch

5. **Betriebssystem**

☐Nur Aufzucht ☐Aufzucht & Mast ☐Ferkelproduzent & Aufzucht ☐Kombinierter Betrieb

Betriebsgröße 6. Anzahl der Zuchtsauen/Rasse (Genetik): ________/___________________

7. Herkunft des Samens: ☐ Eberstation ☐ Sonstige: __________

8. Remontierungsrate: ____________ 9. Eigenremontierung: ☐Ja ☐Teilweise ☐Nein

10. Lebendgeborene Ferkel/Wurf: _____ 11. Abgesetzte Ferkel/Sau/Jahr: _______

Produktionsdaten

12. Produktions/Einstall**rhythmus** ☐Ja ☐Nein

☐Wochen-Rhythmus ☐2-Wochen Rhythmus ☐3-Wochen Rhythmus

☐4-Wochen Rhythmus ☐5-Wochen Rhythmus ☐Andere: __________

13. **PRRS-Status:**

☐Unbekannt ☐Positiv ☐Unverdächtig

☐Geimpft ☐Ungeimpft

14. Wurden im Laufe des letzten Jahres größere betriebliche **Änderungen** (Stallrenovierung, Stallneubaut, Fütterung, Haltung, Management, Reinigung & Desinfektion.) vorgenommen? Wenn ja, welche?

____

____

____

____

____

2. Säugezeit

15. Welche gesundheitlichen Probleme bestanden in den letzten zwölf Monaten bei **Saugferkeln**?

☐Saugferkeldurchfall ☐Lebensschwache Ferkel ☐Andere:___________________________

☐Ferkelruß ☐Husten/Niesen ☐Keine

2.1. Saugferkeldurchfall

16. An welchem **Lebenstag** trat Saugferkeldurchfall vor allem auf?

_____ Lebenstag

17. Wenn ja: waren vermehrt **Jungsauenwürfe** betroffen?

☐Ja ☐Nein

18. Trat der Saugferkeldurchfall vermehrt bei **bestimmten Gruppen/Stallbereichen** auf?

☐Ja ☐Nein

19. Wenn ja, in welchen Bereichen / bei welchen Gruppen?

____

2.2. Versetzen

20. Wurden in den letzten zwölf Monaten Saugferkel versetzt?

☐Nein ☐Ja

21. **Wohin** wurden Ferkel versetzt:

☐Ammensauen ☐Rescue Decks ☐Wurfausgleich ☐Sonstige:___

22. **Welche Ferkel** wurden versetzt:

☐Die Größten ☐Die Kleinsten ☐Ausgeglichen ☐Anders:_____

23. **Welche Nachteile** sehen Sie im Versetzen von Saugferkeln?

____

24**. Bis wann** wurde versetzt?

☐12 Stunden nach der Geburt ☐24 Stunden nach der Geburt

☐2. Lebenstag ☐7. Lebenstag ☐> 7. Lebenstag

25**.** Bei wie viel **Prozent aller Sauen** einer Abferkelgruppe wurden Ferkel versetzt?

☐ <10% ☐ 10 – 30% ☐ 30 – 50% ☐ > 50%

2.3. Prästarter

26. Wurde in den letzten zwölf Monaten Prästarter in der Saugferkelphase verabreicht?

☐Ja ☐Gelegentlich ☐Nie

27. Wenn, ab welchem Lebenstag: _____ Lebenstag (= ______ Tage vor dem Absetzen)

28. Welcher Prästarter wurde verabreicht (**genaue Produktbezeichnung / Firma**):

____

2. Säugezeit

29. **Komponenten (Inhaltsstoffe & Makronährstoffe)** des Prästarters:

____

____

30. Wurde Prästarter auch **nach dem Absetzen** noch mitverfüttert?

☐Ja ☐Nein

31. Wenn ja, für wie viele Tage?

_________________ Tage nach dem Absetzen

32. Wurden zusätzlich andere Futtermittel während der Saugferkelphase angeboten:

☐ Keine ☐ Milchaustauscher ☐ Müsli ☐Sonstige: _______________

33. Wenn ja, bitte um Angabe des/der **Produktnamen/s**:________________________________

Eingesetzt ab: _________________________ Tage vorm Absetzen; Eingesetzt wer:________________

3. Absetzmanagement

3.1. Absetzmanagement

34. **Alter** der Ferkel zum Absetzzeitpunkt:

______________ Tage

35. **Zu/Verkauf** von **Babyferkeln:**

☐ Weder noch ☐ Babyferkel werden verkauft ☐ Babyferkel werden zugekauft

36. Ungefähre Anzahl der verkauften / zugekauften Ferkel pro Absetzgruppe: ________ / _______

37. Zum Absetzzeitpunkt war das **gesamte** Abteil (der ganze Raum) immer **ausnahmslos** unbesetzt:

☐Ja ☐Nein

38. Wie viele Tage/Stunden **steht** das Abteil zwischen zwei Belegungen **leer**: _______________

39. **Durchschnittsgewicht** zum Absetzzeitpunkt: _________kg ☐Geschätzt ☐Gemessen

40**. Wurfweises** Absetzen?
(alle Ferkel eines Wurfes werden zum selben Zeitpunkt in dieselbe Bucht abgesetzt)

☐Ja ☐Nein ☐ Ja, nur die Kleinsten werden gruppiert ☐Sonstiges:______

41. Wenn ja, **wie viele Würfe** kommen gemeinsam in dieselbe Bucht? ____________ Würfe

42. Wenn nein, wonach wurden/werden die Tiere beim Absetzen **sortiert?**

☐Größe/Gewicht ☐Geschlecht ☐Anders:________

43. **Rücksetzen** von Kümmerern:

☐Ja ☐Gelegentlich ☐Nein

44. Wurden in den letzten zwölf Monaten in der Aufzucht **Neugruppierungen** durchgeführt:

☐Ja ☐Gelegentlich ☐Nein

3. Absetzmanagement

45. Was passierte beim Absetzen mit besonders schwachen/kleinen Ferkeln?

☐Sie kommen in dieselbe Bucht wie die anderen abgesetzten Ferkel.
☐Schwache Ferkel werden gruppiert und kommen in eine separate Bucht im selben Abteil.
☐Schwache Ferkel werden gruppiert und kommen in ein anderes Abteil.
☐Sonstiges: _____________

3.2. Räumliche Voraussetzungen

46. **Aufstallungsart(en)** in der Aufzucht:

☐Außenstall ☐Außenklimastall ☐Flat Deck (geschlossen)

47. Art des **Bodens** und dessen Beschaffenheit in der Aufzucht:

☐Vollspaltenböden ☐Teilspaltenböden ☐Planbefestigt
☐Metall ☐Kunststoff ☐Beton ☐Holz ☐Andere:_______

48. Vorhandene **Strukturelemente** der Buchten:

☐Kisten/Abdeckungen ☐Einstreu ☐Keine ☐Sonstige: ___________________

49. Welche Art(en) von **Beschäftigungsmaterial** wird (werden) in der Aufzucht angeboten?

☐Raufutter (Stroh, Heu, etc.) ☐Seile ☐Holz ☐Kunststoffspielzeug
☐Erde, Torf etc. ☐Bälle ☐Ketten ☐Andere: ______________

50. Art der **Heizung** in der Aufzucht:

☐Raumheizung ☐Zonenheizung ☐ Andere & Besonderheiten: ____________
☐Bodenheizung ☐Wandheizung

51. **Wärmeerzeugung**:
☐Warmwassererzeugung ☐Warmlufterzeuger

52. Auf welche **Zieltemperatur** wird im Absetzabteil vor dem Absetzen geachtet?

_____________ °C ☐Auf keine

53. Anzahl der **Absetzabteile (Kammern)** im Betrieb (Abteil = eine räumliche Einheit):

_____________ / Betrieb

54. Anzahl der **Absetzbuchten/Abteil**:

_____________ / Abteil 1 (_____________ / Abteil 2 _____________ / Abteil 3)

55. Anzahl der **Würfe/Absetzbucht** (Problembucht):

_____________ / Bucht

56. Anzahl der **Tiere/Absetzbucht** (Problembucht):

_____________ / Bucht

57. Bleiben die Ferkel nach dem Absetzen bis zum Verkauf/Einstallung in die Mast in dieser Bucht

☐ Ja ☐ Nein, alle werden am _____ Tag nach dem Absetzen umgestallt
☐ Nein, ca. _____% der Ferkel /Bucht kommen ______ Tage nach dem Absetzen in eine andere Bucht

4. Fütterung

4.1. Absetzstarter

58. Bekamen/bekommen die Ferkel zum Zeitpunkt des Absetzens ein eigenes **Absetzstarter**?
☐Ja ☐Nein, es wird dasselbe Aufzuchtfutter über die gesamte Periode gefüttert

59. Ab welchem Tag bekommen die Ferkel den Absetzstarter _______ Tage **vor/nach** dem Absetzen

60. Der Absetzstarter wird für ________ Tage alleine eingesetzt und danach für ____ Tage verschnitten

61. **Hofeigene** Anteile des Absetzstarters: __________________________ ☐Keine

62. **Zugekaufte** Anteile des Absetzstarters (**Konzentrat**/Firma): _________________

63. Anteile (%) des Absetzstarters: Mais: _____% Gerste: _____ % Präparat: __________ %
Präparat 2: _______ % Faserergänzer _______ %, Weizen: _________%,Andere________%

64**. Futtermittelanalyse** des Absetzstarters

**Nährstoffe (Absetzfutter)**	
Anteil der Trockenmasse (%)	%
Rohfasergehalt (g/kg TM)	g/kg TM
Rohproteingehalt (g/kg TM)	g/kg TM
Lysingehalt (g/kg TM)	g/kg TM
Cystein/Methionin (g/kg TM)	g/kg TM

65. **Futterzusätze (Säuren, Probiotika, pflanzliche Zusätze)**

	**Präparat**	**Dosierung** & **Applikationsart**	**Beginn** (Tage vor/nach dem Absetzen)	**Dauer** der Anwendung
**Säuren**				
**Probiotika/Präbiotika**				
**Kräutermischungen**				

66. Andere **Maßnahmen**, die getroffen wurde, um Absetzdurchfall zu verhindern

Kohle				
Impfung („*E. coli* – Ferkelschluckimpfung“)				
Andere				

4. Fütterung

67. Wie oft tritt **Absetzdurchfall seit der Umsetzung** dieser Maßnahmen, wenn in der Vergangenheit versuchsweise auf Zinkoxid verzichtet wurde noch auf?

☐ nicht versucht ☐ nie ☐ selten ☐ gelegentlich ☐oft

68**. Art** der Fütterung

☐Trockenfutterautomat ☐Breiautomat ☐Flüssigfütterung ☐Spotmix
☐Längströge ☐ Rundträge ☐Sonstige:__________

☐mit Hand ☐ automatisch

69. Werden zum Absetzen andere **Maßnahmen** getroffen, um die **Futteraufnahme** der Ferkel nach dem Absetzen **anzuregen**?

☐Ja ☐Nein

70. Wenn ja, welche?____

6.2. Aufzuchtfutter

71. Das Aufzuchtfutter 1 wird ab dem ____ **Tag** nach dem Absetzen eingesetzt

72**. Art** der Fütterung

☐Trockenfutterautomat ☐Breiautomat ☐Flüssigfütterung ☐Spotmix
☐Längströge ☐ Rundträge ☐Sonstige:__________

73. **Hofeigene** Anteile des Aufzuchtfutters: ___________________________ ☐Keine

74. **Zugekaufte** Anteile des Aufzuchtfutters (**Präparat**/Firma): _________________,______________

75. **Rezeptur** (%) des Aufzuchtfutters: Mais: _____%, Gerste: _____ % , Weizen: _______ %,

Soja: _______ %, Präparat 1:_______, Präparat 2 _______ %, Faserergänzer: _______ %

Andere Inhaltsstoffe ______________(%),_____________(%),___________(%)

76**. Futtermittelanalyse** des Aufzuchtfutters

**Nährstoffe (Aufzuchtfutter)**	
Anteil der Trockenmasse (%)	%
Rohfasergehalt (g/kg TM)	g/kg TM
Rohproteingehalt (g/kg TM)	g/kg TM
Lysingehalt (g/kg TM)	g/kg TM
Cystein/Methionin (g/kg TM)	g/kg TM

77. **Besonderheiten** im Fütterungsmanagement (Übergangsfutter etc.):

____

5. Absetzdurchfall

5.1. Einsatz von Zinkoxid

79. Wurde in den letzten zwölf Monaten Zinkoxid eingesetzt? ☐Ja ☐Nein

80. Wenn ja, **welche Tiere** wurden behandelt?

☐Einzeltiere ☐Die ganze Bucht ☐Das ganze Abteil ☐Die gesamte Aufzucht

81. Bei **wie viel Prozent der Absetzgruppen** wurde Zinkoxid eingesetzt?

☐<25% ☐25-50% ☐50-75% ☐>75%

82. Auf welcher Grundlage wurde die **Entscheidung zur Behandlung** gefällt?

☐ Absetzdurchfall stellt im Betrieb ein chronisches Problem dar. Deswegen wird **standardmäßig** therapiert.
☐ Über den Einsatz einer Therapie wird **individuell** entschieden, da die Symptome nicht bei allen Absetzgruppen auftreten.
☐ Eine Behandlung erfolgt ausschließlich nach einem Erregernachweis im **Labor**.
☐ Eine Behandlung erfolgt ausschließlich nach einem Nachweis krankmachender *E. coli* im Labor.

83. Welches **Präparat** wurde verwendet:

☐ Reines Zinkoxid ☐ Kombinationspräparat mit Colistinsulfat

84. **Wie** wurde das Zinkoxid eingesetzt?

☐Fütterung ☐Sonstiges:___________________________

85. In welcher **Dosierung** wurde Zinkoxid zugesetzt (kg/t): ___________________

86. **Ab welchem Tag** vor/nach dem Absetzen wurde es eingesetzt? ______________

87. Für die **Dauer** von wie vielen Tage wurde es eingesetzt?

________________ Tage + _________ Tage verschnitten

5.1.1. Hintergrund zu Zinkoxid

Wir bitten Sie um die Beantwortung folgender Aussagen.

88. Der Einsatz von Zinkoxid kann zur Entwicklung von **Antibiotikaresistenzen** führen.
☐ Richtig ☐ Falsch

89. Zinkoxid ist das ideale Mittel zur Behandlung von **Zinkmangelerscheinungen.**
☐ Richtig ☐ Falsch

90. Der Einsatz von Zinkoxid wird ab 2022 von der Europäischen Union **verboten**.
☐ Richtig ☐ Falsch

5.2. Absetzdurchfall

91. Bestand in den letzten zwölf Monaten ein **Problem** mit Absetzdurchfall (> 10% der Absetzgruppen)?
☐Ja, trotz des Einsatzes von Zink ☐Ja, da auf Zinkoxid verzichtet wurde
☐Nein, wenn Zinkoxid eingesetzt wurde ☐Nein

92. Wie viel **Prozent der Absetzgruppen** waren in den letzten zwölf Monaten von Absetzdurchfall betroffen?

☐<25% ☐25-50% ☐50-75% ☐>75%

5. Absetzdurchfall

93. Wurde ***E. coli*** in den Absetzgruppen im Kot/Darm im **Labor** nachgewiesen?

☐Nicht getestet ☐Nein ☐Ja, ohne Resistenztest ☐Ja, mit Resistenztest

94. **Am wievielten Tag nach dem Absetzen** trat der Durchfall meistens auf?

______________ Tage nach dem Absetzen

95. **Wie lange** dauerte der Durchfall der Absetzgruppe im Schnitt?

☐1-3 Tage ☐4-7 Tage ☐>7 Tage

Welche anderen Probleme bestehen in der Aufzucht? ☐ Kümmern ☐Husten ☐Streptokokken ☐Gelenksentzündungen ☐Sonstige: ________________

5.3. Antibiotische Behandlung

96. Wurde aufgrund eines Bestandsproblemes mit Absetzdurchfall ein Antibiotikum nach erfolgtem Erregernachweis inkl. Resistenztest regelmäßig eingesetzt? ☐ Ja ☐ Nein

97. Wenn ja, **welche Tiere** wurden behandelt?

☐Einzeltiere ☐Die ganze Bucht ☐Das ganze Abteil ☐Die gesamte Aufzucht

98. Bei **wie viel Prozent der Absetzgruppen** wurde in den letzten zwölf Monaten Antibiotika eingesetzt?

☐<25% ☐25-50% ☐50-75% ☐>75%

99. Wenn ja, welches Präparat?
☐ Enteroxid ☐ OTC ☐Amoximix ☐Sonstige:__________________

100. **Wie** wurde das Antibiotikum verabreicht?

☐Fütterung ☐Trinkwasser ☐Einzeltierbehandlung ☐Sonstiges:____________

101. **Ab welchem Tag** vor/nach dem Absetzen wurde es eingesetzt? ___________________

102. Für die **Dauer** von wie vielen Tagen wurde es eingesetzt?

__________________Tage + ____________ Tage verschnitten

103. Welches Therapeutikum wurde in den letzten zwölf Monaten zur Therapie von **stark** an Absetzdurchfall **erkrankten** Tieren gewählt, bzw. im **Einzelfällen**, da Absetzdurchfall kein Bestandsproblem darstellt und eine Therapie nur in Einzelfällen notwendig war. Name des **Präparates**:

☐Baytril® ☐Marbocyl® ☐Sonstige: _______________________

104. **Wie** wurde das Antibiotikum verabreicht?

☐Fütterung ☐Trinkwasser ☐Einzeltierbehandlung ☐Sonstiges:____________

105. Für die **Dauer** von wie vielen Tagen wurde es eingesetzt?__________________Tage

6.Trinkwasser

6.1. Trinkwasserquelle:

106. ☐ Ortswasser ☐ Hofeigen

107. Wurde im Zuge dieser letzten Trinkwasseruntersuchung eine **erhöhte Keimanzahl** nachgewiesen?

☐ Ja ☐ Nein

108. Was war im Zuge der letzten **chemischen** Trinkwasseruntersuchung erhöht?

☐ Eisen ☐ Kalk ☐ Mangan ☐ Keine Auffälligkeiten ☐ Sonstiges:________

109. Befinden sich **Brauchwasserbehälter** auf dem Betrieb?

☐ Ja ☐ Nein

6.2. Trinkwasserleitungen

110. Wie oft wurden in den letzten zwölf Monaten die **Leitungen gereinigt**?

☐ Nie ☐ Vor jeder Neubelegung ☐ Durchgehend ☐ Anderes Regime:_________

111. Wie oft wurden in den letzten zwölf Monaten die **Leitungen desinfiziert**?

☐ Nie ☐ Vor jeder Neubelegung ☐ Durchgehend ☐ Anderes Regime:_________

112. **Womit** wurde desinfiziert?

☐ Chlordioxid ☐ VirkonS® ☐ UV ☐ Sonstiges: ______________

113. Welches **Leitungssystem** wird für die Aufzucht verwendet?

☐ Nur Ringleitungssystem ☐ Leitungssystem mit Kurzschlüssen

114. **Durchmesser** der Rohrleitungen in der Aufzucht (geschätzt):

☐ < 1/2 Zoll ☐ 1/2 Zoll ☐ 3/4 Zoll ☐ 1 Zoll ☐ > 1 Zoll

115. Werden die Leitungen vor jeder Neubelegung so lange **gespült**, bis **frisches Wasser** nachkommt?

☐ Ja ☐ Nein

6.3. Tränken:

116. Art der Tränken: ☐ Nippeltränken ☐ Schalentränken ☐ Sonstige:_____________

117. Werden die **Nippel extra gereinigt & desinfiziert**? ☐ Ja ☐ Nein

7. Reinigung & Desinfektion

7. Reinigung & Desinfektion

7.1. Reinigung der Aufzucht

118. Wie oft wurde die Aufzucht **gereinigt**?

☐Vor jeder Neubelegung ☐Nie ☐Sonstiges:___________

119. **Wer** reinigt?

☐Eigenreinigung ☐Firma: _________________

120. **Womit** wurde gereinigt?

☐Kaltwasser ☐Lauwarmes Wasser ☐Heißes Wasser

☐Hochdruckreiniger ☐Wasserschlauch ☐**Trocken** (Besenrein)

121. Produktname des **Seifenmittels**:

_________________________________ ☐ Nur mit Wasser gereinigt

122. Wie lange wurde **eingeweicht**?

____

7.2. Desinfektion der Aufzucht

123. Wann wurde die Aufzucht **desinfiziert**?

☐Vor jeder Neubelegung ☐Nie ☐Sonstiges:_________________

124. **Zeitabstand** zwischen Reinigung und Desinfektion:

____

125. Wurde vor der Desinfektion darauf penibel geachtet, ob alles **trocken** ist?

☐Ja ☐Nein

126. Name des zur Desinfektion verwendeten **Präparates**:

____

127. Wurde im Zuge der Desinfektion die **Lüftung** in den leeren Abteilen abgeschaltet?

☐Ja ☐Nein

128. Wurde nach Aufbringen des Desinfektionsmittels darauf geachtet, dass die **Flächen feucht** bleiben?

☐Ja ☐Nein

129. Wurde berücksichtigt, dass das verwendete Mittel zur adäquaten Desinfektion eine bestimmte **Mindesttemperatur** benötigt?

☐Nein ☐Ja, aber praktisch kaum durchführbar

☐Ja, deshalb wird die Konzentration angepasst ☐Ja, deshalb wird die Temperatur erhöht.

130. In welcher **Konzentration**/ welchem **Gebrauchsvolumen** wurde das Desinfektionsmittel aufgebracht**:** _______________________________________

131. Wurde die **Gesamtoberfläche**, auf der das Desinfektionsmittel aufgebracht wird **berechnet**?

7. Reinigung & Desinfektion

☐Nein ☐Ja und Volumen/Konzentration des Desinfektionsmittels wurden dieser angepasst

☐Ja, allerdings wurden Volumen/Konzentration ihr nicht angepasst

132. Dauer des **Einwirkens** des Desinfektionsmittels**: ___________________**

7.3. Personal & Gerätehygiene in der Aufzucht

133. Befinden sich vor den Absetzabteilen **Stiefeldesinfektionswannen**?

☐Ja ☐Nein

134. Mit welchem **Präparat:**______________________________________

135. Wie oft werden diese Wannen **gewechselt**? alle _________________

136. Verwendung von **separatem Schuhwerk** für die Aufzucht:☐Ja ☐Nein

137. **Eigene Geräte** (Treibbretter etc.), die ausnahmslos in der Aufzucht verwendet werden:

☐Ja ☐Nein

7.4. Allgemeine Betriebshygiene (auf alle Stallbereiche bezogen)

138. Anzahl der am Betrieb **regelmäßig arbeitenden Personen**: ________

139. **Umkleidemöglichkeit** vorhanden

☐Ja ☐Nein

140. Adäquate **Hygieneschleuse** vorhanden

☐Ja ☐Nein

141. **Betriebseigene Kleidung**

☐Ja ☐Nein

142. **Einduschen** vor Betreten des Stalles

☐Für alle ☐Nur betriebsfremde Personen ☐Niemand

143. **Schadnagerbelastung**:

☐Gering ☐Mittelgradig ☐Stark

144. **Fliegenbelastung**:

☐Gering ☐Mittelgradig ☐Stark

8. Sauen, Impfungen & Parasitenbekämpfung

8.1. Andere gesundheitliche Probleme(Fieber, **MMA**, Klauen, Gelenke, Ausfluss)

145.Welche gesundheitlichen Probleme traten bei Sauen auf?

____

____

____

____

8.2. Impfungen:

146. **Sauenimpfungen**

	Ja	Impfstoff	Zeitpunkt
PRRSV			
Parvo/Rotlauf			
Influenza			
PCV-2			
E. coli/Clostridien			
Stallspezifische Impfstoffe			

147**. Ferkelimpfungen**

	Ja	Impfstoff	Zeitpunkt
PRRSV			
PCV-2			
Mycoplasma hyopneumoniae			
Lawsonia intracellularis			
Ödemkrankheit			
Stallspezifische Impfstoffe			
Andere Ferkelimpfungen			

8.3. Parasitenbekämpfung:

148. Welche **Altersgruppe(n)**? ☐Aufzuchtferkel ☐Mastschweine ☐Zuchtsauen

149. **Wann** (Ferkel) vor/nach dem Absetzen :____________________ Tage_____ dem Absetzen

150. Welches **Präparat** wurde eingesetzt: _______________________

151. Regime – Sauen: ____________ ☐Terminorientiert ☐Produktionsorientiert

152. **Toltrazuril/Kokzidienprophylaxe**:

☐Ja ☐Nein **Wann**: _____________ Lebenstag

Präparat: ________________ **Wie**: ☐Oral ☐In den Muskel

8. Sauen, Impfungen & Parasitenbekämpfung

Messungen im Stall

153. **Größe** von zwei Buchten, in denen sich Ferkel mit Absetzdurchfall befinden (in Meter x Meter):

Bucht 1: ____________________ Bucht 2: ____________________

154. Anzahl der **Nippeltränken/Absetzbucht** (Eigenmessung in einer Problembucht):

Bucht 1: _________________

155. Die **Fließgeschwindigkeit** der Nippeltränken (Messung an zwei Nippeln derselben Bucht) ist subjektiv:

Hgr. erhöht Ggr. erhöht Passend Ggr. vermindert Hgr. vermindert Kein Fluss

156. Höhe dieser zwei **Nippeltränken** (Messung in derselben Bucht wie vorhin):

Nippel 1: _________________ Nippel 2: _________________

78. Gesamte Fressplatzlänge der Futterautomaten/Tröge in einer Bucht:

____
